# A Case of Intragenic Recombination Dramatically Impacting the Phage WO Genetic Diversity in Gall Wasps

**DOI:** 10.3389/fmicb.2021.694115

**Published:** 2021-06-25

**Authors:** Dao-Hong Zhu, Cheng-Yuan Su, Xiao-Hui Yang, Yoshihisa Abe

**Affiliations:** ^1^Laboratory of Insect Behavior and Evolutionary Ecology, College of Life Science and Technology, Central South University of Forestry and Technology, Changsha, China; ^2^College of Life Science, Hunan Normal University, Changsha, China; ^3^Faculty of Social and Cultural Studies, Kyushu University, Fukuoka, Japan

**Keywords:** Cynipidae, gall wasp, horizontal transfer, multiple infections, phage WO, recombination, *Wolbachia*

## Abstract

The phage WO was characterized in *Wolbachia*, a strictly intracellular bacterium causing several reproductive alterations in its arthropod hosts. This study aimed to screen the presence of *Wolbachia* and phage WO in 15 gall wasp species from six provinces of southern China to investigate their diversity and prevalence patterns. A high incidence of *Wolbachia* infection was determined in the gall wasp species, with an infection rate of 86.7% (13/15). Moreover, seven species had double or multiple infections. All *Wolbachia*-infected gall wasp species were found to harbor phage WO. The gall wasp species infected with a single *Wolbachia* strain were found to harbor a single phage WO type. On the contrary, almost all species with double or multiple *Wolbachia* infections harbored a high level of phage WO diversity (ranging from three to 27 types). Six horizontal transfer events of phage WO in *Wolbachia* were found to be associated with gall wasps, which shared identical *orf7* sequences among their respective accomplices. The transfer potentially took place through gall inducers and associated inquilines infected with or without *Wolbachia*. Furthermore, 10 putative recombination events were identified from *Andricus hakonensis* and *Andricus* sp2, which harbored multiple phage WO types, suggesting that intragenic recombination was the important evolutionary force, which effectively promoted the high level of phage WO diversity associated with gall wasps.

## Introduction

*Wolbachia* are maternally inherited endosymbiotic bacteria belonging to the family Anaplasmataceae that infect arthropods and filarial nematodes (Werren, 1997; Stouthamer et al., 1999). The symbiont is extremely widespread among arthropods, and probably infects about half of all terrestrial arthropod species (Hilgenboecker et al., 2008; Zug and Hammerstein, 2012; Weinert et al., 2015). *Wolbachia* manipulates its host’s reproduction by inducing several phenotypes, such as cytoplasmic incompatibility, parthenogenesis, feminization of genetic males, and male-killing (Werren et al., 2008). Bacterial viruses (bacteriophages or phages) are the most abundant organisms in the biosphere and constitute a significant force in bacterial genome evolution (Hendrix et al., 1999; Bordenstein and Wernegreen, 2004). *Wolbachia* phage particles were first observed in the *Wolbachia* infection of *Culex pipiens* by Wright et al. (1978). Subsequently, Masui et al. (2000, 2001) characterized the phage WO, a λ phage-like temperate phage, from the *Wolbachia* strain wTai, infecting *Teleogryllus taiwanemma*. They indicated that the phage WO could be either lysogenic and integrated into the *Wolbachia* chromosome, or lytic and free in the cytoplasm. As a consequence of reductive evolution, mobile DNA elements have often been shown to be rare or absent from obligate intracellular bacteria (Moran and Plague, 2004; Bordenstein and Reznikoff, 2005). However, polymerase chain reaction (PCR) amplification of the minor capsid gene *orf7* showed that the phage WO infected about 90% of supergroups A and B of *Wolbachia* from various arthropod groups (Bordenstein and Wernegreen, 2004; Gavotte et al., 2007). Moreover, nearly all sequenced *Wolbachia* genomes, except those acting as obligate mutualists, harbored prophage WO (Gavotte et al., 2007; Kent and Bordenstein, 2010; Metcalf and Bordenstein, 2012). Considering the wide distribution of *Wolbachia*, phage WO might be one of the most abundant phage lineages in arthropods.

Phage WO is believed to be a dynamic element having a significant impact on the genomic evolution of *Wolbachia* (Wu et al., 2004). As in other prokaryotes, the integration and transformation of prophage are considered major sources of *Wolbachia* lateral gene acquisition (Bordenstein et al., 2006). Phage WO can mediate lateral gene transfer between *Wolbachia* strains, regardless of whether the transferred genes originate from *Wolbachia* or other unrelated bacteria (Ishmael et al., 2009; Wang et al., 2016). They can also regulate the numbers of their host bacteria by inhibiting their replication or inducing cell lysis (Bordenstein et al., 2006). Furthermore, several studies have suggested that phage WO possibly is crucial in *Wolbachia*-induced cytoplasmic incompatibility in insect hosts (Saridaki et al., 2011; LePage et al., 2017; Shropshire et al., 2018). Mutation, recombination, and genome segment reassortment during replication might mediate genetic changes in viruses (Domingo, 2010). A phage genome can be divided into functional units or modules (each one responsible for head or tail formation, lysis, lysogeny, and so forth), which can be mixed by segment reassortment with other phages (Hatfull, 2008). Insertion sequences are frequently found in phage WO genomes and are considered to be a major factor driving these recombinations (Wu et al., 2004; Klasson et al., 2009). The nucleotide sequence of the minor capsid gene *orf7* from the wKueA1 strain of *Wolbachia* is chimeric, and the population genetic analysis has confirmed the occurrence of intragenic recombination events (Bordenstein and Wernegreen, 2004). Furthermore, based on metagenomic analysis, Bordenstein and Bordenstein (2016) demonstrated that genes with eukaryotic homology were constituents of the phage WO, implying lateral gene transfers between bacteriophage/prophage and animal genomes. However, the molecular evolution of phage WO has received far less attention compared with the impact on their bacterial host genome evolution.

Gall wasps (Cynipidae) are a phytophagous group of the superfamily Cynipoidea (Hymenoptera), which usually form structurally complex plant galls on different plant organs. They are the second most species-rich group of gall inducers after the gall midges (Diptera: Cecidomyiidae), with about 1,400 described species (Ronquist et al., 2015). In addition to true gall formers, the Cynipidae also include phytophagous inquilines, which live inside the galls of other species. Several studies have revealed *Wolbachia* infection in diverse cynipid species with high infection rates (Plantard et al., 1998; Abe and Miura, 2002; Rokas et al., 2002; Zhu et al., 2007; Yang et al., 2013; Hou et al., 2020; Zhao et al., 2021), and some gall wasp species show multiple *Wolbachia* infections (Yang et al., 2013; Hou et al., 2020). The results of Yang et al. (2013) suggested a potential possibility of plant tissue-mediated *Wolbachia* horizontal transmission between gall inducers and their associated inquilines. However, no study has reported about phage WO harboring in *Wolbachia*-infected gall wasps. The larvae of gall wasps (sometimes including associated inquilines) feed in completely closed galls. The unique living environment provides a good model to study the transmission and molecular evolution of phage WO within communities. Thus, in this study, the presence of phage WO in 15 gall wasp species collected from six provinces in southern China was detected by employing a PCR-based method with phage WO-specific gene markers so as to determine the phage WO diversity and infection patterns within *Wolbachia*-infected gall wasps. Furthermore, the effects of intragenic recombination and horizontal transmission on phage WO diversity and evolutionary dynamics were also explored.

## Materials and Methods

### Sample Collection and DNA Extraction

The galls of gall wasps were collected from six provinces in southern China during 2012–2020 (Table 1). The galls collected were cage-reared at room temperature in the laboratory of CSUFT. Adult gall wasps were preserved directly in 100% ethanol at −80°C within 2–7 days after emergence until DNA extraction.

**TABLE 1:** Sample information and infection frequency of *Wolbachia* and Phage WO in gall wasps.

**Host plant**	**Location (code)**	**Latitude, longitude**	**Insect species**	***Wolbachia* infect frequency (%)**	**WO infect frequency (%)**	**WO type number**	**Individuals screened**
*Castanea henry*	Qingyuan, Zhejiang (QY)	27°73′N, 119°25′E	*Dryocosmus zhuili*	100 (single)	100	1	20
	Zhenghe, Fujian (ZH)	27°38′N, 118°86’E	*D. zhuili*	100 (single)	100	1	40
	Zhouning, Fujian (ZN)	27°21′N, 119°33′E	*D. zhuili*	100 (single)	100	1	60
*Castanopsis tibetana*	Yanling, Hunan (YL)	26°48′N, 114°04′E	*Dryocosmus liui*	100 (single)	100	1	30
*Quercus fabri*	Jinzhai, Anhui (JZ)	31°64′N, 115°97′E	*Andricus hakonensis*	100 (multiple)	100	9	16
	Changsha, Hunan (CS)	28°21′N, 112°89′E	*A. hakonensis*	100 (multiple)	100	13	40
			*Synergus* sp1	100 (single)	100	1	4
	Suichang, Zhejiang (SC)	28°62′N, 119°31′E	*A. hakonensis*	100 (multiple)	100	18	36
	Qingyuan, Zhejiang (QY)	27°73′, 119°25′E	*A. hakonensis*	100 (multiple)	100	7	40
	Zhouning, Fujian (ZN)	27°21′N, 119°33′E	*A. hakonensis*	100 (multiple)	100	15	48
*Quercus fabri*	Wuhan, Hubei (WH)	30°51′N, 114°52′E	*Andricus* sp1	100 (multiple)	100	1	12
			*Synergus* sp1	100 (single)	100	1	3
*Quercus fabri*	Jinzhai, Anhui (JZ)	31°64′N, 115°97′E	*Andricus* sp2	100 (multiple)	100	2	6
			*Synergus* sp1	100 (single)	100	1	3
	Taihu, Anhui (TH)	30°56′N, 116°07′E	*Andricus* sp2	100 (multiple)	100	7	8
	Wuhan, Hubei (WH)	30°51′N, 114°52′E	*Andricus* sp2	100 (multiple)	100	3	6
	Changsha, Hunan (CS)	28°00′N, 113°01′E	*Andricus* sp2	100 (multiple)	100	8	14
			*Synergus* sp1	100 (single)	100	1	4
*Quercus fabri*	Changsha, Hunan (CS)	28°00′N, 113°01′E	*Andricus* sp3	62.5 (multiple)	50	4	8
*Cyclobalanopsis glauc*	Yanling, Hunan (YL)	26°48′N, 114°04′E	*Plagiotrochus masudai*	100 (two)	90	3	20
*Quercus fabri*	Jinzhai, Anhui (JZ)	31°64′N, 115°97′E	*Aphelomyx glanduliferae*	40 (single)	40	1	20
	Shucheng, Anhui (SHC)	31°35′N, 116°91′E	*A. glanduliferae*	25 (single)	none		4
	Changsha, Hunan (CS)	28°00′N, 113°01′E	*A. glanduliferae*	50 (single)	none		2
*Quercus variabili*	Jinzhai, Anhui (JZ)	31°64′N, 115°97′E	*Latuspina jinzhaiensis*	none	none		40
	Taihu, Anhui (TH)	30°56′N, 116°07′E	*L. jinzhaiensis*	none	none		20
	Wuhan, Hubei (WH)	30°51′N, 114°52′E	*L. jinzhaiensis*	none	none		20
*Quercus chenii*	Changsha, Hunan (CS)	28°13′N, 113°00′E	*Latuspina* sp1	46.7 (multiple)	20	7	30
	Taihu, Anhui (TH)	30°56′N, 116°07′E	*Latuspina* sp1	40 (multiple)	30	5	20
*Quercus variabilis*	Changsha, Hunan (CS)	28°13′N, 113°00′E	*Latuspina* sp2	80 (two)	60	3	10
*Quercus variabilis*	Jinzhai, Anhui (JZ)	31°64′N, 115°97′E	*Cerroneuroterus* sp.	none	none		20
*Quercus fabri*	Jinzhai, Anhui (JZ)	31°64′N, 115°97′E	*Synergus* sp2	100 (single)	100	1	8
	Taihu, Anhui (TH)	30°56′N, 116°07′E	*Synergus* sp2	90 (single)	90	1	10
	Changsha, Hunan (CS)	28°00′N, 113°01′E	*Synergus* sp2	86.6 (single)	86.6	1	30
	Guiding, Guizhou (GD)	26°61′N, 107°23′E	*Synergus* sp2	100 (single)	100	1	6
	Qingyuan, Zhejiang (QY)	27°73′N, 119°25′E	*Synergus* sp2	80 (single)	60	1	10
	Zhouning, Fujian (ZN)	27°21′N, 119°33′E	*Synergus* sp2	75 (single)	62.5	1	16
*Quercus fabri*	Jinzhai, Anhui (JZ)	31°64′N, 115°97′E	*Synergus* sp3	100 (single)	100	1	24
	Taihu, Anhui (TH)	30°56′N, 116°07′E	*Synergus* sp3	100 (single)	100	1	16
	Wuhan, Hubei (WH)	30°51′N, 114°52′E	*Synergus* sp3	100 (single)	100	1	6
	Changsha, Hunan (CS)	28°00′N, 113°01′E	*Synergus* sp3	100 (single)	95	1	40

Adult gall wasps were picked randomly, and total genomic DNA was extracted from each insect using the phenol-chloroform extraction method as described in a previous study (Zhu et al., 2007). The insects were washed with sterile water before DNA extraction to avoid surface contamination. The DNA was resuspended in sterile water and stored at 4°C. This study aimed to screen the quality of each genomic DNA template using nuclear ribosomal DNA internal transcribed spacer 2 gene (Partensky and Garczarek, 2011) and mitochondrial cytochrome coxidase1 (*cox1*) gene (Dyer et al., 2011) using PCR. Poor quality DNA templates were discarded.

### PCR and Sequencing

The samples were first screened for *Wolbachia* infection by PCR amplification. Two primers wsp-81F (5′-TGGTCCAATAAGTGATGAAGAAAC-3′) and wsp-691R (5′-AAAAAT TAAACGCTACTCCA-3′) were used to amplify a portion of the *Wolbachia* surface protein (*wsp*) gene (Zhou et al., 1998). If the amplification failed, another two pairs of primers were used to verify the *Wolbachia* infection: *ftsZ*-F/R for amplification of the *Wolbachia* cell division gene and 16SwolF/R for amplification of the *Wolbachia* 16S RNA gene (O’Neill et al., 1992; Jeyaprakash and Hoy, 2000). WO was screened using the primers WO-F (5′-CCCACATGAGCCAATGACGTCTG-3′) and WO-R (5′-CGTTCGCTCTGCAAGTAACTCCATTAAAAC-3′) to amplify a portion of the capsid protein gene *orf7* (Masui et al., 2000). ddH_2_O was used as a blank control for all amplifications to avoid cross-contamination. The reaction mixture was composed of 1 μL of PrimeSTAR HS DNA Polymerase (Takara Biomedical Technology Co., Ltd, Dalian, China), 10 μL of buffer, 4 μL of dNTPs, 1 μL of each primer, and 2 μL of DNA with water added to achieve a total volume of 50 μL. The amplification was conducted using a C1000 Touch thermal cycler (Bio-Rad, Hercules, CA, United States). The cycling conditions were 98°C for 3 min, 35 cycles of 98°C for 10 s, 50°C–57°C for 30 s, and 72°C for 1 min.

Subsequently, 2.5 μL of the PCR products were run on a 0.8% agarose gel, and electrophoresis was performed using 1× TAE buffer. The gels were stained with GelRed and observed using a gel imaging system. The PCR products were subsequently purified using a TaKaRa MiniBEST Agarose Gel DNA Extraction Kit Ver. 4.0 (Takara Biomedical Technology Co.), and the *wsp* and *orf7* gene fragments were directly sequenced from purified PCR products using PCR primers. The appearance of multiple peaks in a sample at initial sequencing was taken as an indication of multiple infections. The PCR products were then purified using a DNA gene gel extraction kit and ligated directly into the vector, following the manufacturer’s protocols. For each sample, 15–40 independent positive colonies were isolated and cultured in a lysogeny broth medium fortified with ampicillin. Plasmids were extracted and partially sequenced in both directions using an ABI 3730XL DNA sequencer (Applied Biosystems, Foster City, CA, United States) with M13F/R at Wuhan Icongene Co., Ltd.

### Raw Sequence Treatments

Sequence homology analysis was first performed using the BLAST^1^ program online. Genetic distances between all sequence pairs were calculated using Kimura 2-parameter distance model in MEGA 7. Sequences having greater than 1.5% nucleotide diversity in the *orf7* gene were defined as different haplotypes (Chafee et al., 2010). Different sequences were reserved and identical sequences were removed, yielding *orf7* sequences. The sequences have been deposited in GenBank under the following accession numbers: MW98182–MW980306.

### Phylogenetic Analysis

The *orf7* sequences were aligned to relevant sequences previously published on NCBI^2^ with ClustalW in BioEdit (Hall, 1999). Maximum likelihood (ML) was carried out to construct the phylogenetic tree using IQ-Tree 2.1.1 via the online CIPRES Science Gateway portal (Miller et al., 2010). Model selection for the ML analysis was estimated using the Model test v3.7. ML bootstrap values were generated from 1000 bootstrap replicates, under the general time-reversible (GTR) model in which the gamma distribution and invariant sites were estimated from the data (GTR + I + G).

### Recombination Analysis

The individual segment alignments were analyzed using different methods described in the Recombination Detection Program (RDP5) package to detect the evidence of intragenic recombination (Heath et al., 2006). The six recombination detection methods implemented in the RDP5 program for the identification of recombinant sequences and breakpoints were as follows: 3Seq (Martin and Rybicki, 2000), BootScan/rescan recombination test (Martin et al., 2005), GENECONV (Padidam et al., 1999), MaxChi (Smith, 1992), Chimaera (Posada and Crandall, 2001), and the Siscan method (Gibbs et al., 2000). The default settings were used for all methods, and the highest acceptable *P*-value cutoff was set to 0.05.

## Results

### *Wolbachia* and Phage WO Infection Patterns

The galls were collected from southern China, and adult gall wasps of 15 species were obtained. Among these, three species were inquilines (*Synergus* sp1-3), which did not make galls of their own and lived as nest parasites in the galls made by other gall-inducing hosts. Using the diagnostic PCR approach with the *wsp* gene and the phage minor capsid protein gene (*orf7*)-specific primers, a total of 770 wasps of all species obtained for *Wolbachia* and WO infections were screened. The results are listed in Table 1.

Furthermore, 13 out of 15 gall wasp species were infected with *Wolbachia*, and the infection rate of these species was 86.7%. The population infection rates of *Wolbachia*-infected gall wasps ranged from 25 to 100%. Among these, six species were infected with a single *Wolbachia* strain, while the other seven species had double or multiple infections. The samples were tested for *Wolbachia* infection by PCR using specific primers for the *ftsZ* and 16S RNA genes to further confirm that *Latespina jinzhaiensis* and *Cerroneuroterus* sp. were *Wolbachia*-free. The results were all negative.

All *Wolbachia*-infected gall wasp species were found to harbor phage WO. The population infection rates of phage WO ranged from 20 to 100%. Interestingly, the gall wasp species infected with a single *Wolbachia* strain (*Dryocosmus zhuili*, *Dryocosmus liui*, *Aphelomyx glanduliferae*, and *Synergus* sp1–3) were found to harbor a single phage WO type. However, almost all species (including different geographical populations) with double or multiple *Wolbachia* infections harbored diverse phage WO. *Andricus* sp1 was an exception, which had multiple *Wolbachia* infections but carried only one phage type (Table 1). No phage WO was detected in *L. jinzhaiensis* and *Cerroneuroterus* sp. not infected with *Wolbachia.* Although a 273-bp *orf7* sequence was obtained from six insects in the Jinzhai and Wuhan populations of *L. jinzhaiensis*, and the first 217 bp at the 3′-end shared 83% identity with the normal phage WO types, it was a non-coding pseudogene (accession no.: MW980306).

### Phage WO Diversity and Typing

**FIGURE 1:**
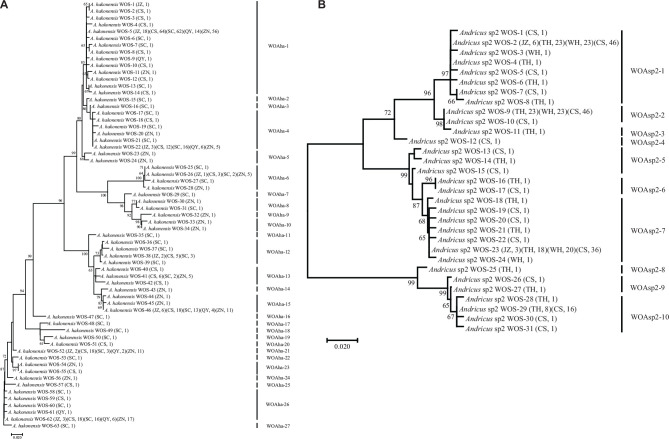
Maximum likelihood tree for phage WO types of *Andricus hakonensis* **(A)** and *Andricus* sp2 **(B)** based on the *orf7* sequences. The letters in parentheses indicate the sampled populations shown in Table 1, and the numbers indicate the number of sequences obtained per population. WOS-1 refers to the serial number. Phage WO types are shown on the right. Numbers above branches are bootstrap values computed from 1,000 replications.

Phage types with similarity on *orf7* DNA sequences larger than 98.5% were defined as identical types according to a previous study (Chafee et al., 2010). For a single *Wolbachia* strain-infected wasp species (*D. zhuili*, *D. liui*, *A. glanduliferae*, and *Synergus* sp1–3) and one species *Andricus* sp1 with multiple *Wolbachia* infections, completely identical *orf7* sequences were obtained from different individuals or/and populations. They harbored only one phage WO type (Table 1). On the contrary, other gall wasp species infected with multiple *Wolbachia* strains harbored phage WO types with a high level of diversity (Figure 1 and Supplementary Figure 1). A total of 493 *orf7* sequences were obtained from five geographical populations of *Andricus hakonensis*, which could be divided into 27 types, WOAha-1–27. Types WOAha-1, WOAha-4, WOAha-15, WOAha-21, and WOAha-26 were found from all five populations, with an abundance rate of 45.8, 9.5, 10.3, 7.3, and 13.0%, respectively. Several types were found with only one *orf7* sequence from one population (Figure 1A). The 318 *orf7* sequences obtained from the four populations of *Andricus* sp2 belonged to 10 phage types, WOAsp2-1–10. Among these, types WOAsp2–1, WOAsp2–2, WOAsp2–7, and WOAsp2–10 were detected from two to four populations, and they accounted for 33.0, 29.2, 26.1, and 8.5% of total sequences, respectively (Figure 1B). Furthermore, *Plagiotrochus masudai*, *Latuspina* sp1, *Latuspina* sp2, and *Andricus* sp3 harbored three, nine, three, and four WO types, respectively (Supplementary Figure 1).

### Phage WO Phylogeny and Horizontal Transfer

**FIGURE 2:**
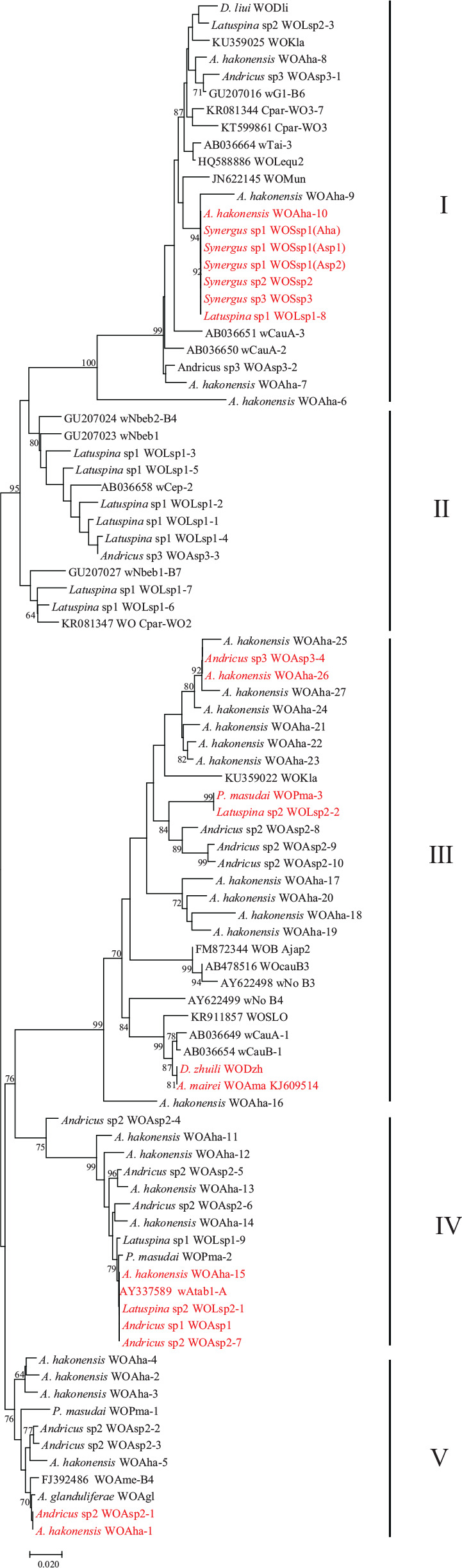
Maximum likelihood phylogenetic tree of the phage WO *orf7* nucleotide sequences found in this study with known phage WO types. Numbers above branches are bootstrap values computed from 1,000 replications. Roman numerals on the right indicate different groups. Red font indicates identical *orf7* sequences among their respective accomplices.

Phylogenetic reconstruction of phage WO *orf7* sequences from gall wasps and other reference insect species was performed using ML methods (Figure 2). According to the phylogenetic tree, the phage WO types clustered into five distinct clades, labeled as groups I–V. The average *orf7* nucleotide divergence levels within and between groups were estimated using the Jukes and Cantor method. The intragroup value was 4.7, 5.1, 7.8, 4.8, and 4.6% in groups I, II, III, IV, and V, respectively, and the intergroup value was 11.9 ± 3.2% (mean ± standard deviation) in average. The groups I–III included the representative phage WO types of their groups retrieved from the gene bank, and the phage WO types from gall wasps tested in the present survey were divided into groups I–V. Although groups IV and V contained ungrouped known types, phage WO from gall wasps could be clustered into new independent branches, which were distinct from groups I–III. Phage WO types of single type harbored in gall wasps belonged to group I (WODli, WOSsp1–3), III (WDzh), IV (WOAsp1), and V (WOAgl). Different types of multiple-infection phage WO types harbored in the same insect species might belong to different groups, for example, those from *A. hakonensis* belonged to groups I, III, IV, and V; from *P. masudai* and *Andricus* sp2 belonged to groups III, IV, and V; from *Latuspina* sp1 belonged to groups I, II, and IV; from *Latuspina* sp2 belonged to groups I, III, and IV; and from *Andricus* sp3 belonged to groups I, II, and III.

No congruence was found between phage WO and its host *Wolbachia* phylogenies, suggesting that phages did not cospeciate with their hosts. Although the phylogenetic relationship between phages and its host *Wolbachia* was not compared, this study provided direct evidence for six horizontal transmission events of phage WO types from gall wasps: (1) WOAha-10 (from *A. hakonensis*), WOSsp1(Aha) (from *Synergus* sp1, inquiline, live inside the galls of *A. hakonensis*), WOSsp1(Asp1) (from *Synergus* sp1, inquiline), WOSsp1(Asp2) (from *Synergus* sp1, inquiline, live inside the galls of *Andricus* sp2), WOSsp2 (from *Synergus* sp2, inquiline), WOSsp3 (from *Synergus* sp3, inquiline), and WOLsp1-8 (from *Latuspina* sp1); (2) WOAsp3–4 (from *Andricus* sp3), and WOAha-26 (from *A. hakonensis*); (3) WOPma-3 (from *P. masudai*) and WOLsp2-2 (from *Latuspina* sp2); (4) WODzh (from *D. zhuili*) and WOAma (from *Andricus mairei*); (5) WOAha-15 (from *A. hakonensis*), WOLsp2-1 (from *Latuspina* sp2), WOAsp1 (from *Andricus* sp1), and WOAsp2–7 (from *Andricus* sp2); (6) WOAsp2–1 (from *Andricus* sp2) and WOAha-1 (from *A. hakonensis*), which shared identical *orf7* sequences among their respective accomplices (Figure 2).

### Intragenic Recombination of *orf7*

**FIGURE 3:**
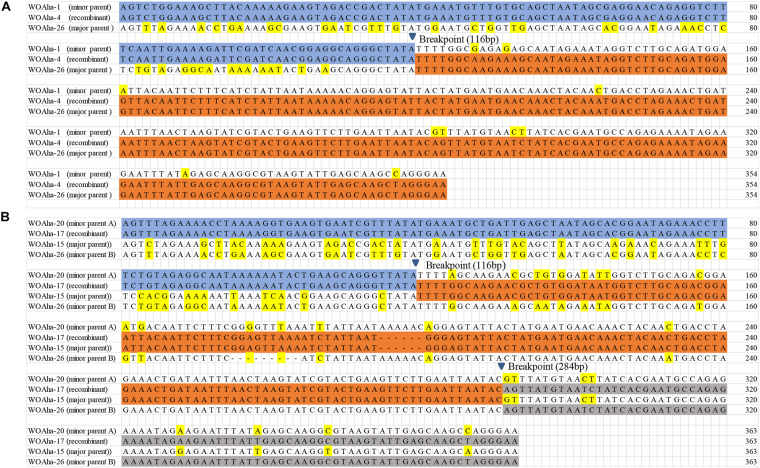
Recombination events of the *orf7* gene **(A)** between WOAha-1 and WOAha-26 resulting in recombinant WOAha-4, and **(B)** among WOAha-20, WOAha-15, and WOAha-26 resulting in recombinant WOAha-17.

The larvae of gall wasps fed in completely enclosed galls, and the occurrence of phage WO diversity in the closed niche provided an ideal sample for obtaining direct evidence of gene recombination. Recombination analysis of the aligned *orf7* sequences was performed using RDP5 programs to understand the extent to which recombination contributed to the diversification of phage WO in gall wasps. In this study, 10 putative recombination events were identified, resulting in new phage types, from *A. hakonensis* to *Andricus* sp2, which harbored diverse phage WOs (Table 2, Figure 3 and Supplementary Figures 2–5).

**TABLE 2:** Recombination analysis of *orf7* gene using six methods implemented RDP5 package in gall wasps harbored with multiple phage WO.

**Insect**	**Recombinant**	**Major parent**	**Minor parent A/B**	**Breakpoint**	**Method**	***P*-value**
***Andricus hakonensis***						
	WOAha-4 (V)*	WOAha-26 (III)	WOAha-1 (V)	116	3Seq	8.61E-10
					BootScan	4.28E-07
					GENECONV	4.31E-06
	WOAha-2 (V)	WOAha-1 (V)	WOAha-26 (III)	298/334	GENECONV	4.23E-04
					BootScan	9.24E-05
	WOAha-2 (V)	WOAha-1 (V)	WOAha-12 (IV)	298/334	BootScan	1.78E-04
					3Seq	8.06E-04
	WOAha-12 (IV)	WOAha-15 (IV)	WOAha-4 (V)	298	3Seq	4.96E-07
					GENECONV	3.03E-04
					MaxChi	8.08E-04
	WOAha-12 (IV)	WOAha-15 (IV)	WOAha-26 (III)	298	3Seq	3.94E-08
					GENECONV	2.47E-05
	WOAha-23 (III)	WOAha-26 (III)	WOAha-16 (III)	298	3Seq	1.03E-05
					MaxChi	3.14E-04
	WOAha-17 (III)	WOAha-20 (III)	WOAha-15 (IV)/WOAha-26 (III)	116/298	3Seq	5.31E-10/4.36E-09
					BootScan	3.28E-09/2.36E-09
					GENECONV	4.31E-09/4.21E-08
					MaxChi	8.86E-08/1.27E-07
	WOAha-7 (I)	WOAha-8 (I)	WOAha-1 (V)/	116/334	3Seq	9.86E-10/6.79E-09
			WOAha-15 (IV)		BootScan	7.53E-09/6.28E-09
					GENECONV	8.12E-09/6.38E-08
					MaxChi	7.94E-08/5.63E-07
***Andricus* sp2**						
	WOAsp2–3 (V)	WOAsp2–7 (IV)	WOAsp2–1 (V)	161	3Seq	3.53E-05
					BootScan	5.96E-05
	WOAsp2–4 (IV)	WOAsp2–7 (IV)	WOAsp2–1 (V)	116/334	3Seq	2.48E-07
					MaxChi	5.11E-05
					Chimera	5.11E-05

*Roman numerals in parentheses refer to the group number of the phage WO type.

In *A. hakonensis*, eight *orf7* gene recombination events were detected (Table 2). Recombinants were obtained in two ways; one major parent and one minor parent or one major parent and two minor parents were recombined into a new phage WO lineage. The former involved six recombination events, while the latter involved two. For example, recombination events between type WOAha-26 and WOAha-1 and among WOAha-20, WOAha-15, and WOAha-26 are shown in Figure 3 (for others, see Supplementary Figures 2–5). Type WOAha-4 was detected as a recombinant by three of the six used methods: 3Seq (*P* < 10^–9^), BootScan (*P* < 10^–6^), and GENECONV (*P* < 10^–5^). The major and minor parents were WOAha-26 and WOAha-1, and the beginning breakpoint was 116 bp. WOAha-17 was detected as a recombinant by four methods: 3Seq (*P* < 10^–9^/10^–8^), BootScan (*P* < 10^–8^/10^–8^), GENECONV (*P* < 10^–5^/10^–7^), and MaxChi (*P* < 10^–4^/10^–6^). The major parent was WOAha-20, and the minor parents were WOAha-15 and WOAha-26; the beginning breakpoint was 116 and 284 bp, respectively. In *Andricus* sp2, two phage WO recombinants were identified, WOAsp2–3 and WOAsp2–4, and their presumed parent types were WOAsp2–1 and WOAsp2–7, respectively (Table 2).

Almost all the parents involved in the recombination events were highly abundant phage lineages. The five phage types (WOAha-1, WOAha-4, WOAha-15, WOAha-21, and WOAha-26) harbored in *A. hakonensis* with a wide distribution and high abundance (Figure 1A); all participated in the recombination as parents and were the main force for the recombination. Similarly, the parents WOAsp2–1 and WOAsp2–7 of recombination were the most abundant types harbored in *Andricus* sp2 (Figure 1B). On the contrary, the abundance of the phage WO recombinant was lower in both *A. hakonensis* and *Andricus* sp2. In addition, WOAha-4 and WOAha-12, which were obtained by recombination, could also be used as parents of recombination to contribute to the diversity of phages, suggesting the frequent occurrence of intragenic recombination. Furthermore, most *orf7* gene recombination events occurred between groups, including group III and V, III, and IV, IV, and V, I and V, and I and IV, except for group III, where recombination between different phage WO types of the same group was found, and almost all recombinants belonged to the same group as their major parents (Table 2).

## Discussion

Although bacteriophages have usually been proven to be rare or lacking in obligate intracellular bacteria (Moran and Plague, 2004; Bordenstein and Reznikoff, 2005), phage WO is widely distributed in various *Wolbachia*-infected insect groups (Bordenstein and Wernegreen, 2004; Gavotte et al., 2007; Kent and Bordenstein, 2010; Metcalf and Bordenstein, 2012; Wang et al., 2016; Kaushik et al., 2019). The present study demonstrated that 86.7% (13/15) gall wasp species were infected with *Wolbachia*, and all *Wolbachia*-infected gall wasps were found to harbor phage WO. From two populations of *L. jinzhaiensis*, a *Wolbachia*-free species, a 273-bp *orf7*-like non-coding pseudogene of phage WO was obtained. It might be considered as the vestige of prophage DNA remaining in the chromosomes of the host insect after a previous lateral gene transfer event, suggesting that *L. jinzhaiensis* might have been infected by *Wolbachia* carrying phage WO. Based on the phylogenetic analysis of the *orf7* sequences from the gall wasps in this study and other reference insect species, the phage WO types were divided into five groups, and each group contained the phage WO harbored in *Wolbachia*-infected gall wasps.

Multiple phage infections, where a *Wolbachia* strain displayed more than one phage type, have been reported in several *Wolbachia* strains (Chauvatcharin et al., 2006; Gavotte et al., 2007). However, most phage-infected *Wolbachia* strains display low numbers of phage types, with 85% showing only one or two different phage types (Gavotte et al., 2007; Tanaka et al., 2009). The findings of the present study indicated that a single type of phage WO was found in seven gall wasp species, which were infected with one strain of *Wolbachia*, except for *Andricus* sp1 with multiple *Wolbachia* infections. On the contrary, other six gall wasp species infected with double or multiple *Wolbachia* strains harbored diverse types of phage WOs: *A. hakonensis* with 27 types, *Andricus* sp2 with 10 types, *Latuspina* sp1 with nine types, *P. masudai* and *Latuspina* sp2 with three types, and *Andricus* sp3 with four types. The presence of multiple *Wolbachia* strains has been documented in several insect species (van Borm et al., 2001; Jamnongluk et al., 2002; Reuter and Keller, 2003; Hou et al., 2020). For gall wasps, a high level of multiple *Wolbachia* infections was found in *A. mukaigawae* and its associated inquiline *Synergus japonicus* with five and eight strains, respectively (Yang et al., 2013), and *Belonocnema treatae* with four strains (Schuler et al., 2018). *Wolbachia* strains were identified by *wsp* gene genetic distance greater than 2%. *P. masudai*, *Latuspina* sp2, and *Andricus* sp3 were found to be infected with three, three, and four *Wolbachia* strains, respectively, and *A. hakonensis*, *Andricus* sp2, and *Latuspina* sp1 were infected with more than ten *Wolbachia* strains (data not shown and will be published in another study). Bacteriophages provide beneficial genes to the bacterial host (Abedon and Lejeune, 2005) or mediate the horizontal transfer of genes (Wommack and Colwell, 2000). Several reports have proven that phage WO can mediate horizontal gene transfer between *Wolbachia* strains (Ishmael et al., 2009; Wang et al., 2016). Therefore, diverse types of phage WOs harbored in the gall wasp species with a high level of multiple *Wolbachia* infections, effectively promoting the molecular evolution of host and increasing *Wolbachia* diversity through mediating the horizontal gene transmission or/and providing beneficial genes.

The absence of an evolutionary correlation between WO and *Wolbachia* phylogenies indicates that many horizontal phage WO transfers have occurred between different *Wolbachia* endosymbionts (Bordenstein and Wernegreen, 2004; Gavotte et al., 2004; Wang et al., 2016). The results of the present study suggested that an abundant horizontal transfer of phage WO in *Wolbachia* was associated with gall wasps. Prophages undergo a lytic phase capable of rupturing bacterial and eukaryotic cell membranes, and phage WO occurs in the extracellular matrix of arthropods. Thus, they might pass through the eukaryote cell wall and then initiate new infections (Masui et al., 2001; Bordenstein et al., 2006; Gavotte et al., 2007). In the closed system of galls, living organisms include gall formers, inquilines, parasitoids, *Wolabchia*, phage WO, and so forth. Gall inducer-inquiline association (Yang et al., 2013) and host-parasitoid association (Hou et al., 2020) are two known routes of horizontal transmission of *Wolbachia* in gall wasps. In three inquilines, *Synergus* sp1 (including individual insects obtained from the galls made by different gall wasps) and *Synergus* sp3 were infected with the same *Wolbachia* strain, while *Synergus* sp2 was infected with another *Wolbachia* strain (Supplementary Figure 6); however, they all carried the same phage WO type. The phage WO type was also detected in *A. hakonensis*. *D. zhuili*, and *A. mairei* infected with different *Wolbachia* strains (Hou et al., 2020), but they carried phage WO with identical *orf7* sequence. Therefore, it was highly likely that phage WOs were transferred in gall wasp species through gall inducer-inquiline association (and host-parasitoid association) with or without *Wolbachia*.

Recombination occurs with both DNA and RNA viruses, and has been viewed as a means to rescue fit viral genomes from low fitness parents or a means to produce highly divergent genomes, resulting in dramatically impact evolution and epidemiology (Chetverin et al., 2005; Domingo, 2010). Bordenstein and Wernegreen (2004) confirmed the recombinogenic nature of phage WO, and, in the case of the capsid protein gene *orf7*, the recombination rate was the fastest reported rate for *Wolbachia* genome. In this study, 10 putative recombination events were identified from *A. hakonensis* and *Andricus* sp2, which harbored multiple phage WO types. The recombinant types and both parent types were all found in the same insect species. This study was novel in providing practical molecular evidence supporting *orf7* gene recombination of phage WO. These results strongly suggested that intragenic recombination was the important evolutionary force, which effectively promoted the high level of phage WO diversity associated with gall wasps (such as *A. hakonensis* and *Andricus* sp2). Furthermore, phage types in almost all groups participated in recombination, and recombination events occurred within or between groups. The phage WO phylogenetic relationship constructed using only the *orf7* gene sequence was not highly reliable due to the frequent occurrence of recombination.

## Data Availability Statement

The datasets presented in this study can be found in online repositories. The names of the repository/repositories and accession number(s) can be found below: https://www.ncbi.nlm.nih.gov/, MW980182-MW980305 and MZ325445-MZ325462.

## Author Contributions

D-HZ and C-YS designed the study, wrote the manuscript, and performed experiments. D-HZ, C-YS, and X-HY performed the analyses. YA identified the gall wasps. YA and X-HY revised the manuscript. All authors contributed to the article and approved the submitted version.

## Conflict of Interest

The authors declare that the research was conducted in the absence of any commercial or financial relationships that could be construed as a potential conflict of interest.

## Footnotes

1 https://blast.ncbi.nlm.nih.gov/Blast.cgi

2 https://www.ncbi.nlm.nih.gov/

## Supplementary Material

Supplementary Figure 1 Maximum likelihood tree for phage WO types of *Plagiotrochus masudai* **(A)**, *Latuspina* sp1 **(B)**, *Latuspina* sp2 **(C)**, and *Andricus* sp3 **(D)** based on the *orf7* sequences. The letters in parentheses indicate the sampled populations shown in Table 1, and the numbers indicate the number of sequences obtained per population. WOS-1 refers to the serial number. Phage WO types are shown on the right. Numbers above branches are bootstrap values computed from 1,000 replications.

Supplementary Figure 2 Recombination events of the *orf*7 gene **(A)** between WOAha-1 and WOAha-26 resulting in recombinant WOAha-2, and **(B)** among WOAha-8, WOAha-1, and WOAha-15 resulting in recombinant WOAha-7.

Supplementary Figure 3 Recombination events of the *orf*7 gene between WOAha-1 and WOAha-12 resulting in recombinant WOAha-2 **(A)**, WOAha-15 and WOAha-4 resulting in recombinant WOAha-12 **(B)**.

Supplementary Figure 4 Recombination events of the *orf*7 gene between WOAha-15 and WOAha-26 resulting in recombinant WOAha-12 **(A)**, WOAha-26 and WOAha-16 resulting in recombinant WOAha-23 **(B)**.

Supplementary Figure 5 Recombination events of the *orf*7 gene between WOAsp2–7 and WOAsp2–1 resulting in recombinant WOAsp2–4 **(A)**, WOAsp2–7 and WOAsp2–1 resulting in recombinant WOAsp2–3 **(B)**.

Supplementary Figure 6 Maximum likelihood phylogenetic tree of the *Wolbachia wsp* nucleotide sequences with single infection found in this study (underlining) with known *Wolbachia* strains from gall wasps.
