# Supplementary material for: A Case of Intragenic Recombination Dramatically Impacting the Phage WO Genetic Diversity in Gall Wasps
Source: Front Microbiol. 2021 Jun 25;12:694115. doi: 10.3389/fmicb.2021.694115 (PMC8279768; doi:10.3389/fmicb.2021.694115)
Supplement: Supplementary Figure 1 — Maximum likelihood tree for phage WO types of Plagiotrochus masudai (A), Latuspina sp1 (B), Latuspina sp2 (C), and Andricus sp3 (D) based on the orf7 sequences. The letters in parentheses indicate the sampled populations shown in Table 1, and the numbers indicate the number of sequences obtained per population. WOS-1 refers to the serial number. Phage WO types are shown on the right. Numbers above branches are bootstrap values computed from 1,000 replications. [file Presentation_1.PPTX]

## Slide 1
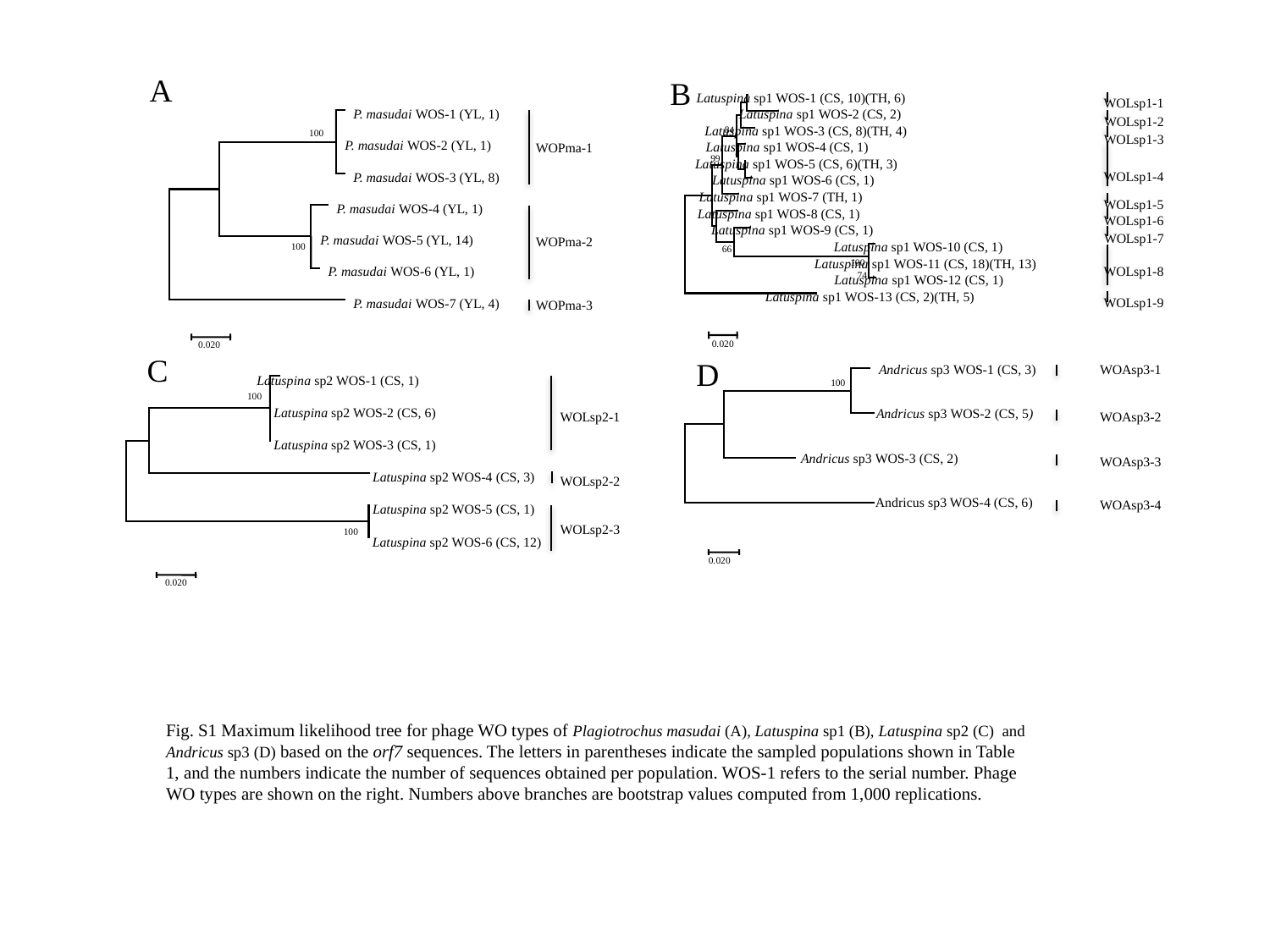

A
B
 P. masudai WOS-1 (YL, 1)
100
 P. masudai WOS-2 (YL, 1)
 P. masudai WOS-3 (YL, 8)
 P. masudai WOS-4 (YL, 1)
 P. masudai WOS-5 (YL, 14)
 P. masudai WOS-6 (YL, 1)
 P. masudai WOS-7 (YL, 4)
0.020
WOPma-1
WOPma-2
100
WOPma-3
WOLsp1-1
 Latuspina sp1 WOS-1 (CS, 10)(TH, 6)
 Latuspina sp1 WOS-2 (CS, 2)
 Latuspina sp1 WOS-3 (CS, 8)(TH, 4)
84
 Latuspina sp1 WOS-4 (CS, 1)
 Latuspina sp1 WOS-5 (CS, 6)(TH, 3)
 Latuspina sp1 WOS-6 (CS, 1)
 Latuspina sp1 WOS-7 (TH, 1)
 Latuspina sp1 WOS-8 (CS, 1)
 Latuspina sp1 WOS-9 (CS, 1)
 Latuspina sp1 WOS-10 (CS, 1)
66
 Latuspina sp1 WOS-11 (CS, 18)(TH, 13)
100
74
 Latuspina sp1 WOS-12 (CS, 1)
 Latuspina sp1 WOS-13 (CS, 2)(TH, 5)
0.020
WOLsp1-2
WOLsp1-3
99
WOLsp1-4
WOLsp1-5
WOLsp1-6
WOLsp1-7
WOLsp1-8
WOLsp1-9
C
D
WOAsp3-1
 Andricus sp3 WOS-1 (CS, 3)
100
 Andricus sp3 WOS-2 (CS, 5)
 Andricus sp3 WOS-3 (CS, 2)
 Andricus sp3 WOS-4 (CS, 6)
0.020
WOAsp3-2
WOAsp3-3
WOAsp3-4
 Latuspina sp2 WOS-1 (CS, 1)
100
 Latuspina sp2 WOS-2 (CS, 6)
 Latuspina sp2 WOS-3 (CS, 1)
 Latuspina sp2 WOS-4 (CS, 3)
 Latuspina sp2 WOS-5 (CS, 1)
 Latuspina sp2 WOS-6 (CS, 12)
0.020
WOLsp2-1
WOLsp2-2
WOLsp2-3
100
Fig. S1 Maximum likelihood tree for phage WO types of Plagiotrochus masudai (A), Latuspina sp1 (B), Latuspina sp2 (C) and Andricus sp3 (D) based on the orf7 sequences. The letters in parentheses indicate the sampled populations shown in Table 1, and the numbers indicate the number of sequences obtained per population. WOS-1 refers to the serial number. Phage WO types are shown on the right. Numbers above branches are bootstrap values computed from 1,000 replications.

## Slide 2
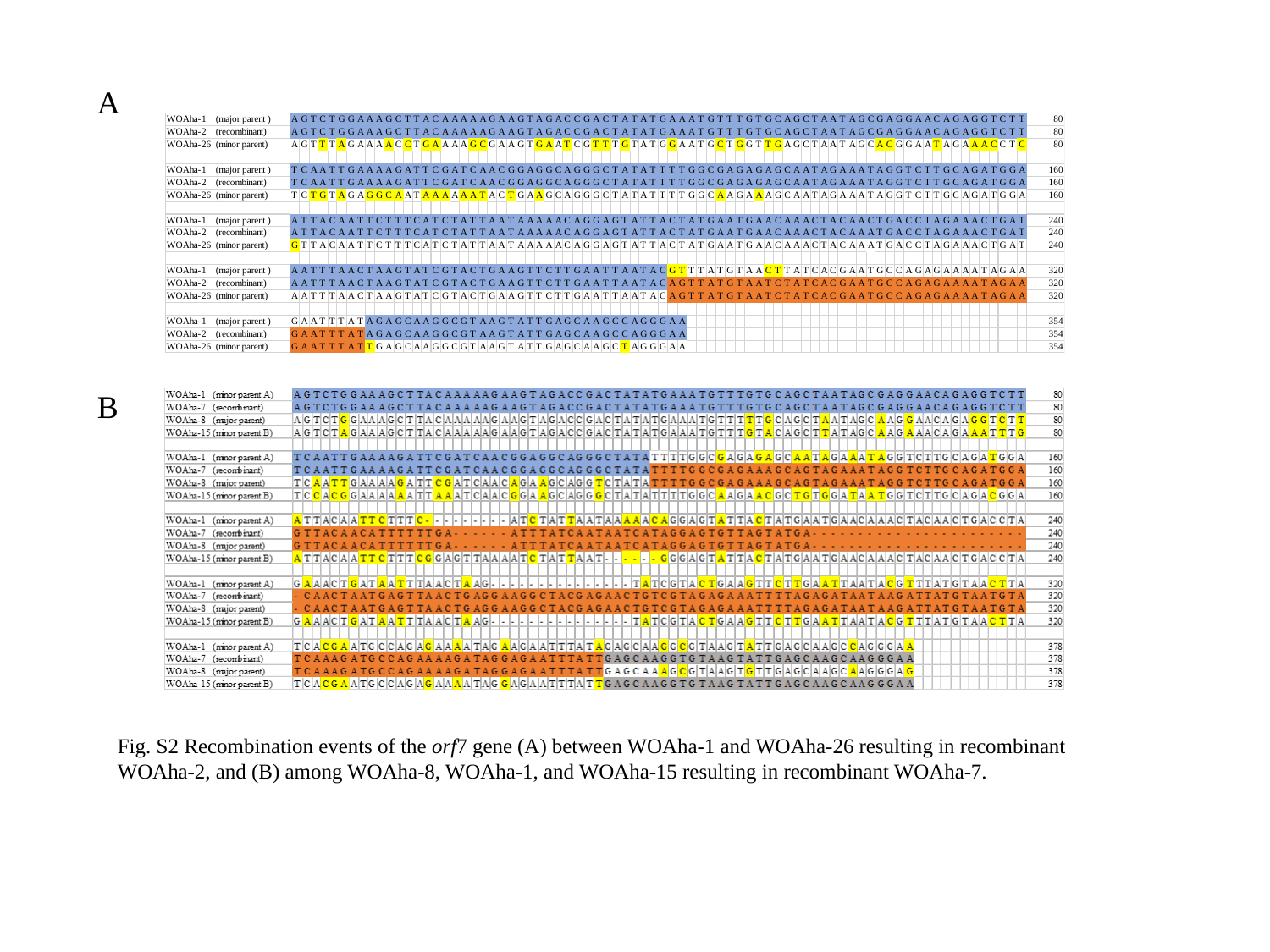

A
B
Fig. S2 Recombination events of the orf7 gene (A) between WOAha-1 and WOAha-26 resulting in recombinant WOAha-2, and (B) among WOAha-8, WOAha-1, and WOAha-15 resulting in recombinant WOAha-7.

## Slide 3
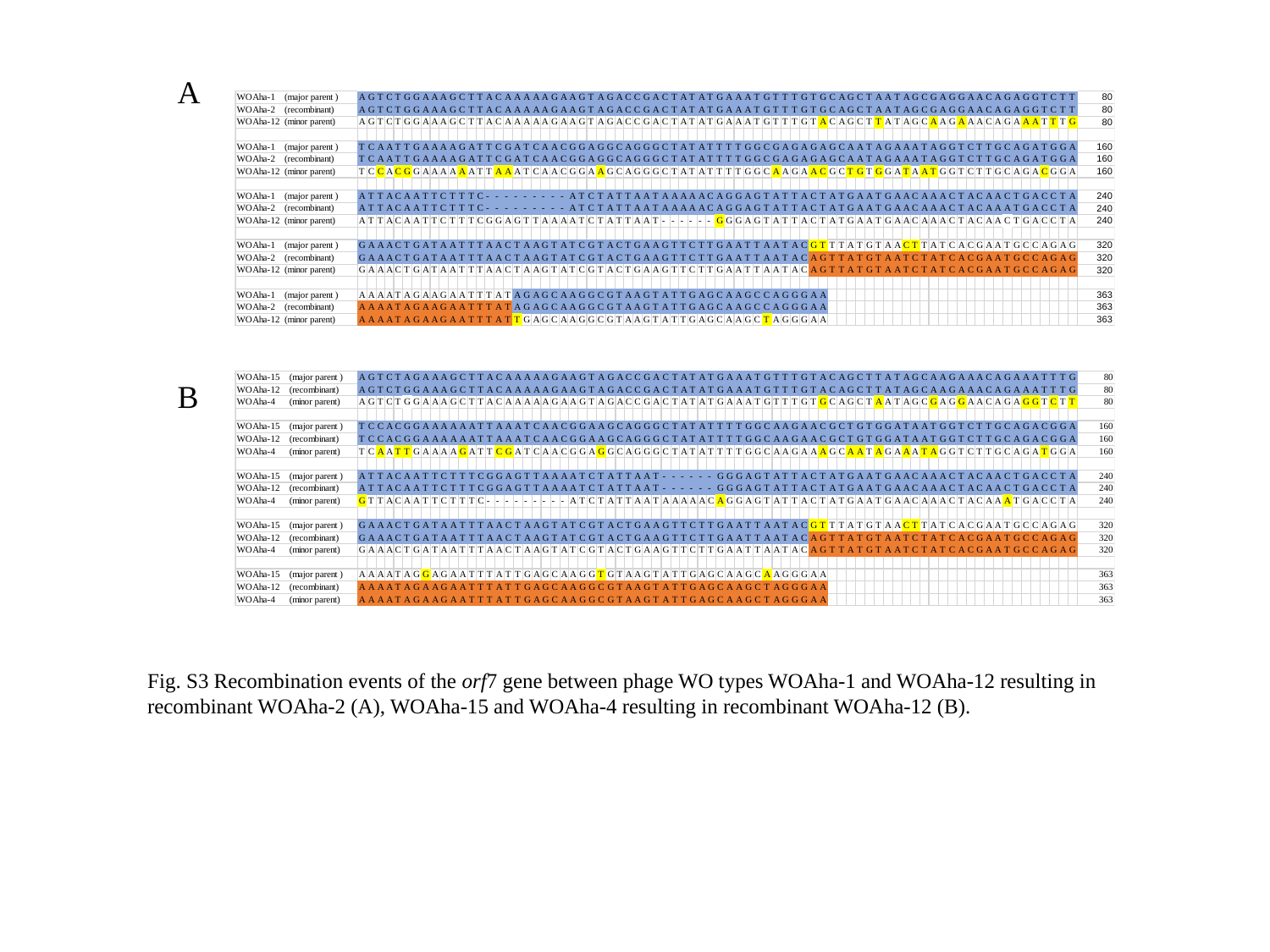

A
B
Fig. S3 Recombination events of the orf7 gene between phage WO types WOAha-1 and WOAha-12 resulting in recombinant WOAha-2 (A), WOAha-15 and WOAha-4 resulting in recombinant WOAha-12 (B).

## Slide 4
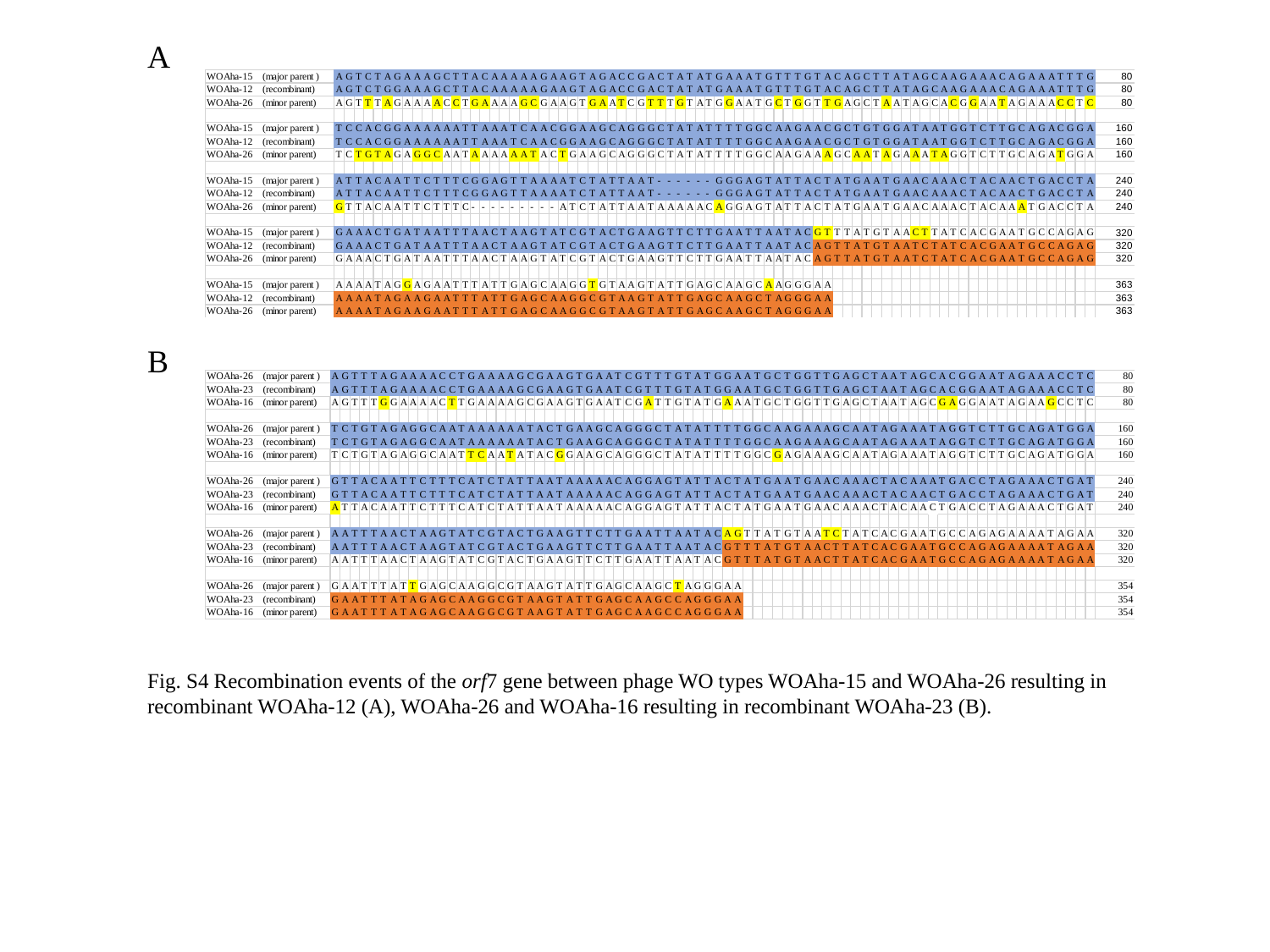

A
B
Fig. S4 Recombination events of the orf7 gene between phage WO types WOAha-15 and WOAha-26 resulting in recombinant WOAha-12 (A), WOAha-26 and WOAha-16 resulting in recombinant WOAha-23 (B).

## Slide 5
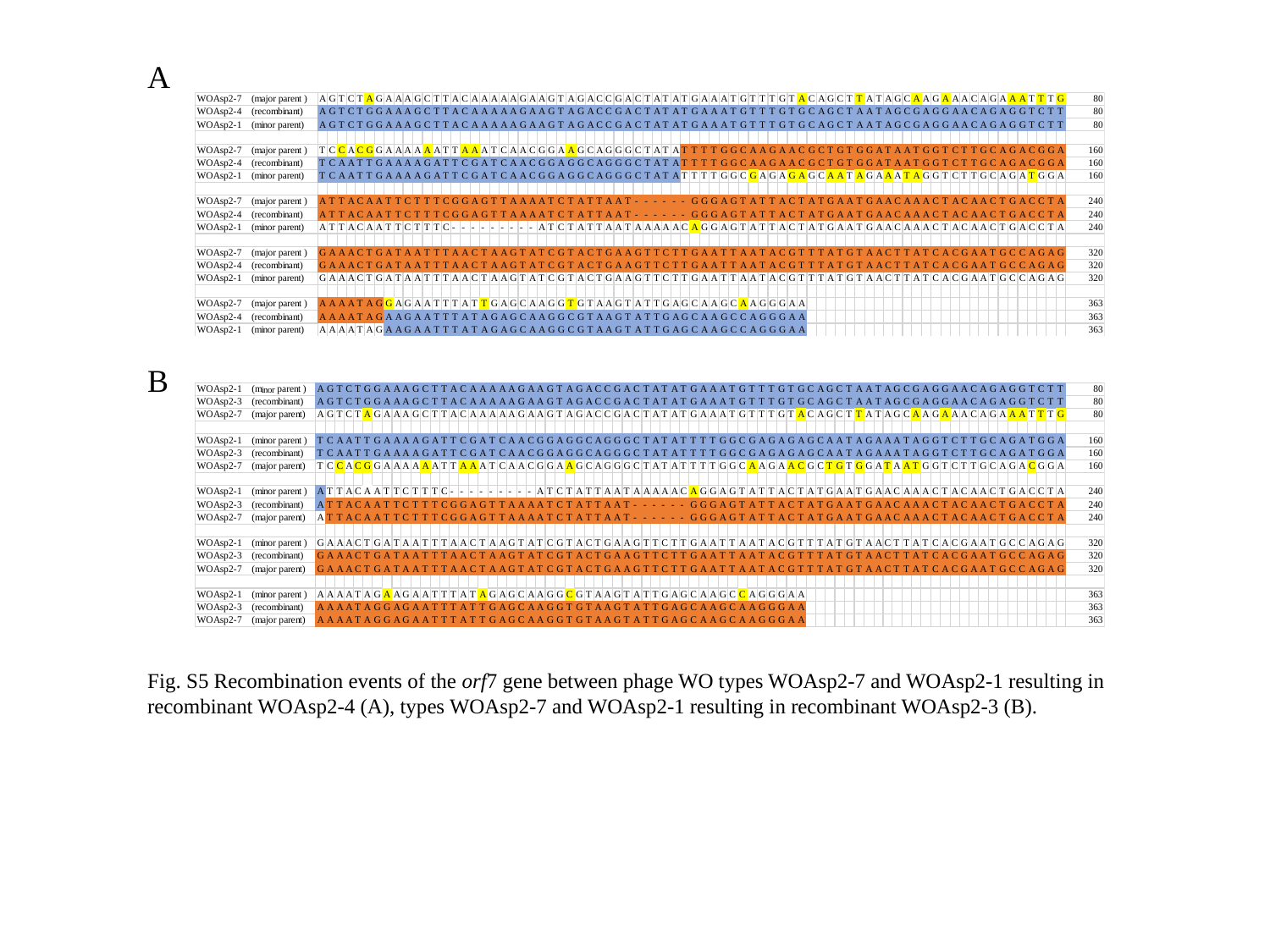

A
B
Fig. S5 Recombination events of the orf7 gene between phage WO types WOAsp2-7 and WOAsp2-1 resulting in recombinant WOAsp2-4 (A), types WOAsp2-7 and WOAsp2-1 resulting in recombinant WOAsp2-3 (B).

## Slide 6
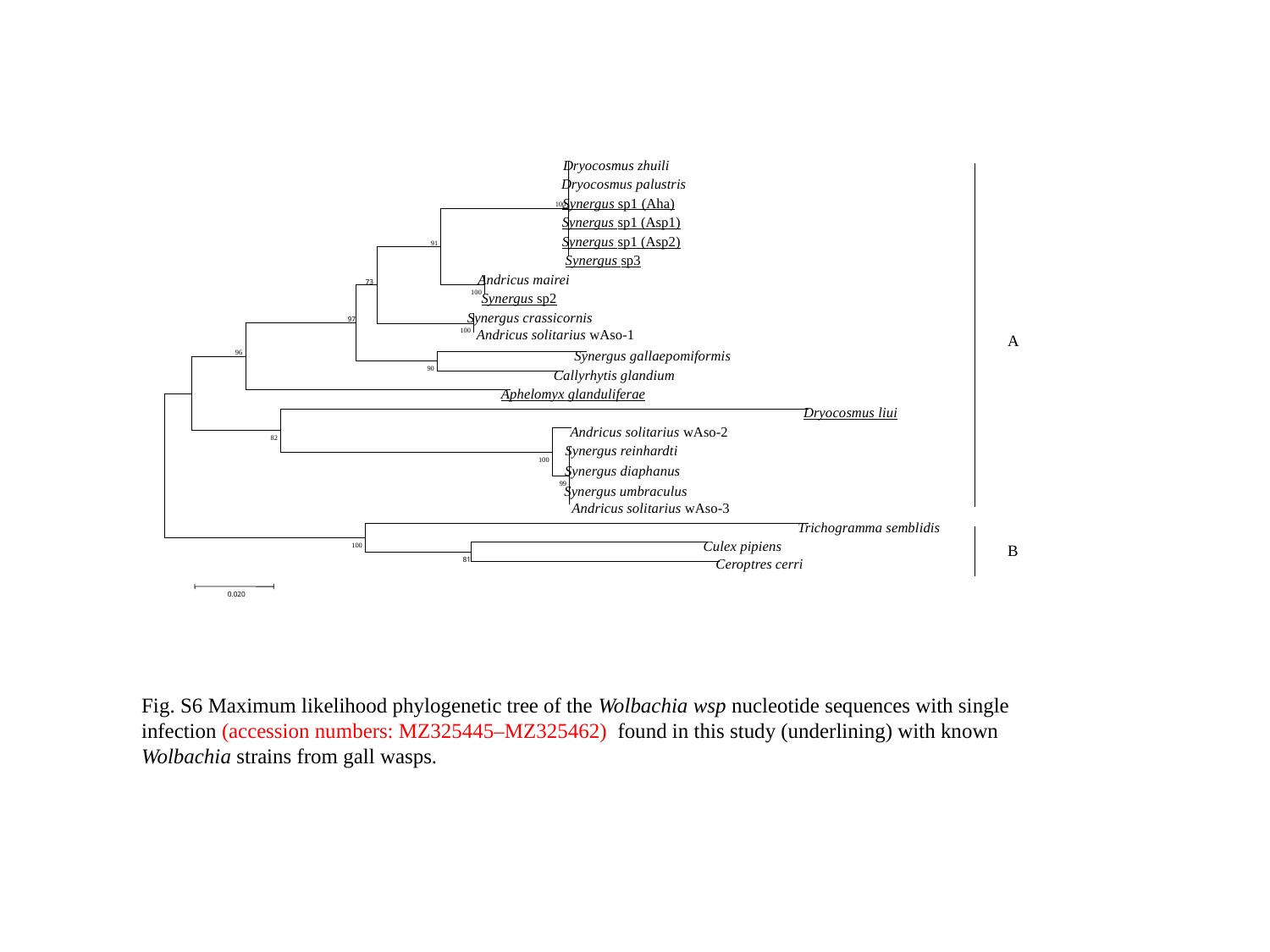

Dryocosmus zhuili
Dryocosmus palustris
 Synergus sp1 (Aha)
100
 Synergus sp1 (Asp1)
 Synergus sp1 (Asp2)
91
 Synergus sp3
Andricus mairei
73
100
 Synergus sp2
Synergus crassicornis
97
Andricus solitarius wAso-1
100
Synergus gallaepomiformis
96
90
Callyrhytis glandium
 Aphelomyx glanduliferae
 Dryocosmus liui
Andricus solitarius wAso-2
82
Synergus reinhardti
100
Synergus diaphanus
99
Synergus umbraculus
 Andricus solitarius wAso-3
Trichogramma semblidis
 Culex pipiens
100
Ceroptres cerri
81
0.020
A
B
Fig. S6 Maximum likelihood phylogenetic tree of the Wolbachia wsp nucleotide sequences with single infection (accession numbers: MZ325445–MZ325462) found in this study (underlining) with known Wolbachia strains from gall wasps.
